# Targeting the microcirculation in resuscitation of patients with septic shock: fluid is superior to norepinephrine

**DOI:** 10.1186/2197-425X-3-S1-A646

**Published:** 2015-10-01

**Authors:** J Xu, Y Yang, H Qiu

**Affiliations:** Southeast University, Zhongda Hospital, Nanjing, China

## Introduction

Our goal was to examine whether fluid improve microcirculation when compared with norepinephrine in responsive septic shock patients despite early goal-directed therapy.

## Methods

This was a single centre prospective study. Patients in septic shock for less than 24 hours despite fluid resuscitation were enrolled. Passive leg raising test was performed to predict fluid responsiveness. Responders were randomizedly divided into two groups, i.e. Resp-FL group and Resp-NE group to maintain usual MAP. In Resp-FL group, patients were treated by fluid until with no response to passive leg raising test, then the MAP was titrated to usual level by NE. In Resp-NE group, doses of NE were adjusted to maintain usual level of MAP directly. Nonresponders were enrolled in Nonresp group, MAP was titrated to usual MAP by norepinephrine.

## Results

Twenty-four patients were enrolled, including 14 responders (7 in Resp-FL group and 7 in Resp-NE group) and 10 nonresponders. Compared with nonresponders, lactate of responders was significantly lower (2.9 ± 2.8 vs. 1.3 ± 0.7 mmol/L, p < 0.05). Compared with Nonresp group, the change of cardiac output was significantly higher in Resp-FL group and Resp-NE group (33.1 ± 28.5 vs. 28.1 ± 16.8%, p < 0.05). Compared with Resp-FL group(TVD: 30.6 ± 10.7%, PVD: 67.8 ± 58.2%, MFI: 61.5 ± 23.4%), the change of microcirculatory parameters were significantly lower of Resp-NE group (TVD: 11.1 ± 18.4%, PVD: 11.8 ± 17.2%, MFI: 15.8 ± 14.5%).

## Conclusions

Responsiveness influenced the choice of resuscitation strategy in early septic shock patients despite early goal-directed therapy. In responders, microcirculatory improvement was found when compared fluid with norepinephrine.

